# *GenomegaMap*: Within-Species Genome-Wide d_N_/d_S_ Estimation from over 10,000 Genomes

**DOI:** 10.1093/molbev/msaa069

**Published:** 2020-03-13

**Authors:** Daniel J Wilson, The CRyPTIC Consortium, Derrick W Crook, Timothy E A Peto, A Sarah Walker, Sarah J Hoosdally, Ana L Gibertoni Cruz, Joshua Carter, Clara Grazian, Sarah G Earle, Samaneh Kouchaki, Alexander Lachapelle, Yang Yang, David A Clifton, Philip W Fowler, Zamin Iqbal, Martin Hunt, Jeffrey Knaggs, E Grace Smith, Priti Rathod, Lisa Jarrett, Daniela Matias, Daniela M Cirillo, Emanuele Borroni, Simone Battaglia, Arash Ghodousi, Andrea Spitaleri, Andrea Cabibbe, Sabira Tahseen, Kayzad Nilgiriwala, Sanchi Shah, Camilla Rodrigues, Priti Kambli, Utkarsha Surve, Rukhsar Khot, Stefan Niemann, Thomas A Kohl, Matthias Merker, Harald Hoffmann, Katharina Todt, Sara Plesnik, Nazir Ismail, Shaheed Vally Omar, Lavania Joseph, Guy Thwaites, Thuong Nguyen Thuy Thuong, Nhung Hoang Ngoc, Vijay Srinivasan, Timothy M Walker, David Moore, Jorge Coronel, Walter Solano, George F Gao, Guangxue He, Yanlin Zhao, Chunfa Liu, Aijing Ma, Baoli Zhu, Ian Laurenson, Pauline Claxton, Anastasia Koch, Robert Wilkinson, Ajit Lalvani, James Posey, Jennifer Gardy, Jim Werngren, Nicholas Paton, Ruwen Jou, Mei-Hua Wu, Wan-Hsuan Lin, Lucilaine Ferrazoli, Rosangela Siqueira de Oliveira, Irena Arandjelovic, Angkana Chaiprasert, Iñaki Comas, Calle Jaime Roig, Francis A Drobniewski, Maha R Farhat, Qian Gao, Rick Ong Twee Hee, Vitali Sintchenko, Philip Supply, Dick van Soolingen

**Affiliations:** Big Data Institute, Nuffield Department of Population Health, Li Ka Shing Centre for Health Information and Discovery, University of Oxford, Oxford, United Kingdom

**Keywords:** d_N_/d_S_, adaptation, natural selection, recombination, big data, parent-dependent mutation

## Abstract

The $d_{N}/d_{S}$ ratio provides evidence of adaptation or functional constraint in protein-coding genes by quantifying the relative excess or deficit of amino acid-replacing versus silent nucleotide variation. Inexpensive sequencing promises a better understanding of parameters, such as $d_{N}/d_{S}$, but analyzing very large data sets poses a major statistical challenge. Here, I introduce *genomegaMap* for estimating within-species genome-wide variation in $d_{N}/d_{S}$, and I apply it to 3,979 genes across 10,209 tuberculosis genomes to characterize the selection pressures shaping this global pathogen. *GenomegaMap* is a phylogeny-free method that addresses two major problems with existing approaches: 1) It is fast no matter how large the sample size and 2) it is robust to recombination, which causes phylogenetic methods to report artefactual signals of adaptation. *GenomegaMap* uses population genetics theory to approximate the distribution of allele frequencies under general, parent-dependent mutation models. Coalescent simulations show that substitution parameters are well estimated even when *genomegaMap*’s simplifying assumption of independence among sites is violated. I demonstrate the ability of *genomegaMap* to detect genuine signatures of selection at antimicrobial resistance-conferring substitutions in *Mycobacterium tuberculosis* and describe a novel signature of selection in the cold-shock DEAD-box protein A gene *deaD/csdA*. The *genomegaMap* approach helps accelerate the exploitation of big data for gaining new insights into evolution within species.

## Introduction

Interpreting patterns of substitution in genetic sequences is a fundamental approach in evolutionary biology. For example, an excess rate of amino acid-replacing *nonsynonymous* substitution compared with silent *synonymous* substitution, quantified by the $d_{N}/d_{S}$ ratio (also denoted $K_{A}/K_{S}$ or *ω*), provides evidence of adaptive change, whereas the reverse pattern, more prevalent in functional protein-coding sequences, provides evidence for purifying selection (e.g., Miyata and Yasunaga 1980; Perler et al. 1980; Nei and Gojobori 1986; Nielsen and Yang 1998). Although the $d_{N}/d_{S}$ ratio has known limitations (see Discussion), it is simple and widely used.

Estimating substitution parameters like $d_{N}/d_{S}$ typically relies on first estimating, or co-estimating, a phylogenetic tree relating the observed sequences. Two major drawbacks commonly arise when 1) recombination is present or 2) sample sizes are large. The first major drawback, often encountered in analyses of within-species variation, is that recombination breaks the assumption of a single phylogeny, and instead generates a network of ancestral relationships in which different genes, and different positions within genes, can have different phylogenetic histories (Schierup and Hein 2000). It is well established that inappropriate application of phylogeny-based methods to recombining data can produce highly misleading biological inferences, including false signals of adaptive evolution in the form of artificially elevated $d_{N}/d_{S}$ (Anisimova et al. 2003; Shriner et al. 2003).

The second major drawback is the computational cost of estimating a phylogeny when the number of sequences becomes large, for example, the 10,209 genomes recently published by CRyPTIC Consortium and 100,000 Genomes Project (2018) that bear witness to the relentless evolution of antimicrobial resistance in tuberculosis. This is a double blow because the cost of evaluating the fit of an individual phylogeny increases at the same time as the number of possible phylogenies explodes (Felsenstein 1973, 1978). Although highly efficient algorithms exist, the problem will become increasingly acute with the steady march toward ever more sequencing.

Wilson and McVean (2006) developed a method, *omegaMap*, to estimate $d_{N}/d_{S}$ in the presence of recombination. Although *omegaMap* avoids the false signals of adaptive evolution suffered by phylogenetic methods, its application to large data sets is limited by the underlying PAC (product of approximate conditionals) approach, whose computational complexity increases quadratically with sample size (Li and Stephens 2003).

In this article, I address these drawbacks with existing methods by introducing *genomegaMap*, a phylogeny-free statistical approach to estimating substitution parameters that implicitly integrates over phylogenetic relatedness using diffusion theory and the coalescent (Wright 1949; Kingman 1982). Since *genomegaMap* interprets codon count information, its computational cost remains constant even as the sample size increases arbitrarily, making it a viable approach for extremely large data sets. The method assumes independence between sites, yet simulations show that the method performs well even when the absence of recombination causes strong linkage disequilibrium. I demonstrate the utility of the method by estimating variation in $d_{N}/d_{S}$ ratios in 3,979 genes sequenced in 10,209 *Mycobacterium tuberculosis* genomes (CRyPTIC Consortium and 100,000 Genomes Project 2018).

## Materials and Methods

### Population Genetics Model

Estimating the $d_{N}/d_{S}$ ratio is a special case of the more general problem of estimating a substitution rate matrix. The Nielsen and Yang (1998) (NY98) codon model assumes that a nonsynonymous substitution occurs at *ω* times the rate of its synonymous counterpart. It is defined by the following substitution rate from codon *i* to *j* (j≠i):

(1) $$\theta_{ij}=\pi_{j}\mu \{\begin{array}{cc} 1 & \text{for}\;\text{synonymous}\;\text{transversion}\\ \kappa & \text{for}\;\text{synonymous}\;\text{transition}\\ \omega & \text{for}\;\text{nonsynonymous}\;\text{transversion}\\ \kappa \omega & \text{for}\;\text{nonsynonymous}\;\text{transition}\\ 0 & \text{otherwise} \end{array},$$

where *ω* is the $d_{N}/d_{S}$ ratio, *κ* the transition:transversion ratio, and *π_j_* the equilibrium frequency of allele *j*. To form a proper rate matrix, the diagonal elements must be defined as $\theta_{ii}=-\sum_{j\neq i}\theta_{ij}$. The scaling constant *μ* is determined by the expected substitution rate, $\theta =\sum_{i}\sum_{j\neq i}\pi_{i}\theta_{ij}$. Following the convention in population genetics, the rate is defined here in units of $2PN_{e}$ generations, where *P* is the ploidy and *N_e_* the effective population size.

*GenomegaMap* estimates substitution parameters by modeling the allele frequency distribution at each site. Analyses of $d_{N}/d_{S}$ within species (e.g., Nielsen and Yang 1998; Wilson and McVean 2006) have implicitly treated selection as a form of *mutational bias*, in which the mutation rate matrix equals the NY98 substitution rate matrix, and fitness differences between individuals are ignored. I follow the convention here (see Discussion). For an alternative approach, see *gammaMap* (Wilson et al. 2011), which separately models mutation and selection.

The distribution of allele frequencies under the simplifying assumptions of a stable and unstructured population, selective neutrality, and *parent independent* mutation, in which the rate of mutation from allele *i* to *j* (*θ_ij_*) depends only on the target allele *j* (so can be written $\theta_{ij}=\theta_{\cdot j}$), is derived from diffusion theory and follows a Dirichlet distribution (Wright 1949; Watterson 1977):

(2) $$p\left(f\right)=\frac{\prod_{j=1}^{K}f_{j}^{\theta_{\cdot j}-1}}{B\left(\theta_{\cdot }\right)},$$

where *f_j_* is the population frequency of allele *j*, *K* is the number of alleles and $B\left(\theta_{\cdot }\right)=\prod_{j=1}^{K}\Gamma (\theta_{\cdot j})/\Gamma (\sum_{j=1}^{K}\theta_{\cdot j})$ is the multivariate beta function.

For more general, *parent-dependent*, mutation models, the distribution cannot be easily calculated. Instead, I employ the approach of Wilson et al. (2011, eq. B1) who approximated the allele frequency distribution as a Dirichlet distribution by conditioning on the identity of the oldest allele *A*:

(3) $$p\left(f|A\right)\approx \frac{\prod_{B=1}^{K}f_{B}^{\;\alpha_{AB}-1}}{B\left(\alpha_{A}\right)},$$

where $\alpha_{AB}=m_{AB}/m_{AA}$ and *m_AB_* is the probability of sampling an allele *B* conditional on having sampled allele *A* in a sample of size two, calculable using the coalescent as

$$m_{AB}=\int_{0}^{\infty }{\left\{e^{\theta t}\right\}}_{AB}e^{-t}dt$$

(4) $$=\sum_{k=1}^{K}\frac{V_{Ak}V_{kB}^{\left\{-1\right\}}}{1-D_{kk}},$$

where $\theta =\mathrm{VD}V^{-1}$ is the eigendecomposition of the substitution rate matrix. This approximation, which in principle allows any Markovian substitution process to be fitted, is motivated by a low mutation rate assumption and therefore expected to work best when the expected number of substitutions per site is small.

Assuming random sampling, the conditional allele count distribution is Multinomial-Dirichlet distributed:

Pr(x|A)=∫Pr(x|f)p(f|A)df

(5) $$=\left(\begin{array}{c} n\\ x \end{array}\right)\frac{B\left(x+\alpha_{A}\right)}{B\left(\alpha_{A}\right)},$$

where *x_j_* is the number of times allele *j* was counted, *n* the sample size and Pr(x|f) represents the multinomial distribution. The identity of the oldest allele *A* is then averaged over to obtain the likelihood for the allele counts at a site:

(6) $$\mathrm{Pr}\left(x\right)=\sum_{A=1}^{K}\pi_{A}\mathrm{Pr}\left(x|A\right).$$

The coarsest approximation made by *genomegaMap* is independence between sites, which is motivated by the benefits it confers with the rest of the model: 1) The computational complexity is constant irrespective of sample size, whereas the likelihoods in phylogenetic and PAC models increase linearly and quadratically with sample size, respectively. 2) Missing data can be handled easily because the sample size need not be the same from site-to-site. 3) No haplotype information is required.

### Statistical Inference

*GenomegaMap* uses Bayesian inference for parameter estimation. Three models of variation in *ω* within individual genes were implemented. In the *independent codon* model, the prior distributions on *ω* are independent across codons, so no information is shared about the parameters along the alignment. In the *sliding window* (or piecewise constant) model, adjacent codons share the same value of *ω* with probability $1-p_{\omega }$. This shares information between codons within a “block” of identical *ω*’s and has a smoothing effect on the point estimates (Wilson and McVean 2006). In the constant model, *ω* is assumed constant along the alignment so information is shared across all codons.

Parameters were estimated by Markov chain Monte Carlo (MCMC) using previously published Metropolis–Hastings moves. Scalar parameters (*ω*, *κ*, and *θ*) were updated using log-uniform proposal distributions. For the sliding window model, block boundaries were updated with a geometric proposal whereas blocks were split and merged using reversible jump moves (Wilson and McVean 2006, Appendix B). The equilibrium codon frequencies π were fixed to be uniform (*porB3* analysis only) or to match the empirical codon frequency distribution among 10,209 *M. tuberculosis* genomes (CRyPTIC Consortium and 100,000 Genomes Project 2018).

### Simulations

I performed simulations to test the performance of *genomegaMap* under two scenarios. In the Unlinked simulations, every codon was simulated independently, in keeping with the assumption of *genomegaMap*. In the Clonal simulations, all codons were completely linked, maximally violating this assumption of *genomegaMap*. For each scenario, I simulated 100 data sets of 334 codons in 10,000 individuals. The parameters were simulated independently for each data set from log-normal distributions with (2.5%, 97.5%) quantiles of (0.05, 5) for *ω*, (1, 9) for *κ*, and (0.001, 0.1) for *θ*. *ω* was assumed constant along the sequence. Codon frequencies were simulated from the empirical frequency distribution. For each simulated data set, parameters were estimated using as priors the same distributions used to simulate *ω*, *κ*, and *θ*. Under these conditions, the 95% credibility intervals (CIs) should include the true parameters in 95% of simulations, if the approximate likelihood performs optimally (Dawid 1982). For each analysis, I ran two independent MCMC chains of 10,000 iterations.

### Analysis of *Neisseria meningitidis porB3*

To compare *genomegaMap* with *omegaMap*, I re-analyzed 23 of 79 *porB3 N. meningitidis* sequences of Urwin et al. (2002) comprising the *carriage study* subset of Wilson and McVean (2006). Columns in the alignment with any indels were removed to aid the comparison because *omegaMap* handles them differently. I assumed an exponential prior distribution with mean 1.0 for *ω* and improper log-uniform priors for *κ* and *θ*. I assumed a sliding window model for variation in *ω* along the gene, with a mean block length of $p_{\omega }^{-1}=30$ codons. For both *genomegaMap* and *omegaMap*, I ran two independent MCMC chains of 500,000 iterations. Trace plots were compared visually with assess convergence. 1,000 iterations of burn-in were removed. Chains were merged to obtain final results.

### Analysis of 10,209 *M. tuberculosis* Genomes

CRyPTIC Consortium and 100,000 Genomes Project (2018) collected and whole-genome sequenced 10,209 *M. tuberculosis* samples from 16 countries across six continents comprising strains enriched for antimicrobial resistance and unenriched strains collected for routine clinical diagnostics. They mapped all genomes to the H37Rv reference genome (Cole et al. 1998) (GenBank accession number NC_000962.2). I downloaded the alignment of every genome to H37Rv and combined these to create a multiple sequence alignment for each of the 3,979 CDSs in the GenBank annotation, ignoring insertions relative to H37Rv and masking nonsense mutations.

Inference of *ω*, *κ*, and *θ* for an individual gene can be improved by gleaning information from other genes. Often this is implemented through a hierarchical model, for example, estimating a distribution for the selection parameters across all sites in all genes (Wilson et al. 2011). However, hierarchical modeling requires sophisticated techniques for simultaneously analyzing thousands of genes across a high performance computing cluster. Instead, I mimicked a hierarchical model heuristically by training a prior for *ω*, *κ*, and *θ* using an alignment of 334 codons randomly chosen from the 3,979 genes. For this preliminary analysis, I employed an exponential hyperprior with mean 1.0 for *ω*, imposing a single block across the alignment, and improper log-uniform hyperpriors for *κ* and *θ*, running two MCMC chains for 10,000 iterations. This produced posterior means of –0.79, 1.2, and –2.9 and standard deviations of 0.20, 0.21, and 0.15 for log ω,  log κ, and log θ, respectively.

I used these results to form priors for the analyses of the 3,979 individual genes by assuming log-normal distributions, multiplying the standard deviation parameters by 10 for *ω* and 3.2 for *κ* and *θ* to avoid overinformative priors. This produced a prior median and (2.5%, 97.5%) quantiles of 0.45 (0.0098, 21) for *ω*, 3.2 (0.90, 12) for *κ*, and 0.057 (0.023, 0.14) for *θ*. I analyzed the data under a mixture of two models with equal prior probability: 1) the sliding window model with mean block length $p_{\omega }^{-1}=33$ codons and 2) the independent codon model. For each gene, I ran two independent MCMC chains of 500,000 or 1,000,000 iterations for the two models, respectively, with 50,000 iterations removed as burn-in. The chain lengths were chosen from preliminary runs where convergence and burn-in were assessed visually. The median run times per gene per chain were 233 and 172 min for the two models, respectively. I used the harmonic mean estimate of the Bayes factors to merge the results for each gene and to obtain posterior model probabilities.

### Software and Data Availability

*GenomegaMap* is available as a Docker container and C++ source code from https://hub.docker.com/r/dannywilson/genomegamap and https://github.com/danny-wilson/genomegaMap. The following data are available: codon counts for every annotated CDS https://doi.org/10.6084/m9.figshare.7599020.v1 and a summary of the Bayesian analysis at the gene level (supplementary table S1, Supplementary Material online) and codon level https://doi.org/10.6084/m9.figshare.10329311.

## Results

### General Performance of *GenomegaMap*

**Fig. 1.**
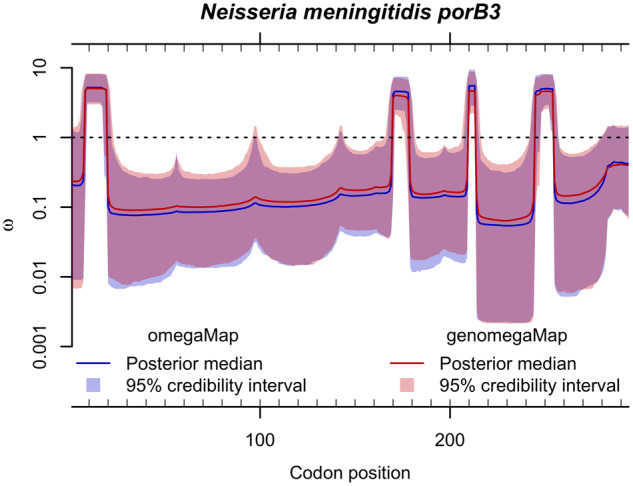
Comparison of *omegaMap* and *genomegaMap* estimates of the $d_{N}/d_{S}$ ratio *ω* along the *porB3* outer membrane protein gene of *Neisseria meningitidis*. Solid lines and shaded regions show the point estimates (posterior medians) and 95% credibility intervals, respectively, for *omegaMap* (in blue) and *genomegaMap* (in red). The *genomegaMap* runs were 4.9 times faster for these 23 sequences at 92 min each.

The motivation for developing *genomegaMap* came from the observation that *omegaMap* estimates of substitution parameters, including the $d_{N}/d_{S}$ ratio *ω*, were not strongly affected by the exact value of the recombination rate, as long as it was nonzero. This observation is reflected in the comparison of the analyses of the *N. meningitidis porB3* gene (fig. 1), for which the point estimates and 95% CIs of *ω* were almost identical between *omegaMap* and *genomegaMap*, even though the latter assumes codons are independent, that is, unlinked. Although the results were near-identical, the *genomegaMap* point estimates and 95% CIs were slightly more conservative, in the sense that they were closer to the prior expectation of *ω *= 1. These results suggest that substitution parameters are well estimated within species when sites are assumed independent, despite the presence of linkage disequilibrium.

**Fig. 2.**
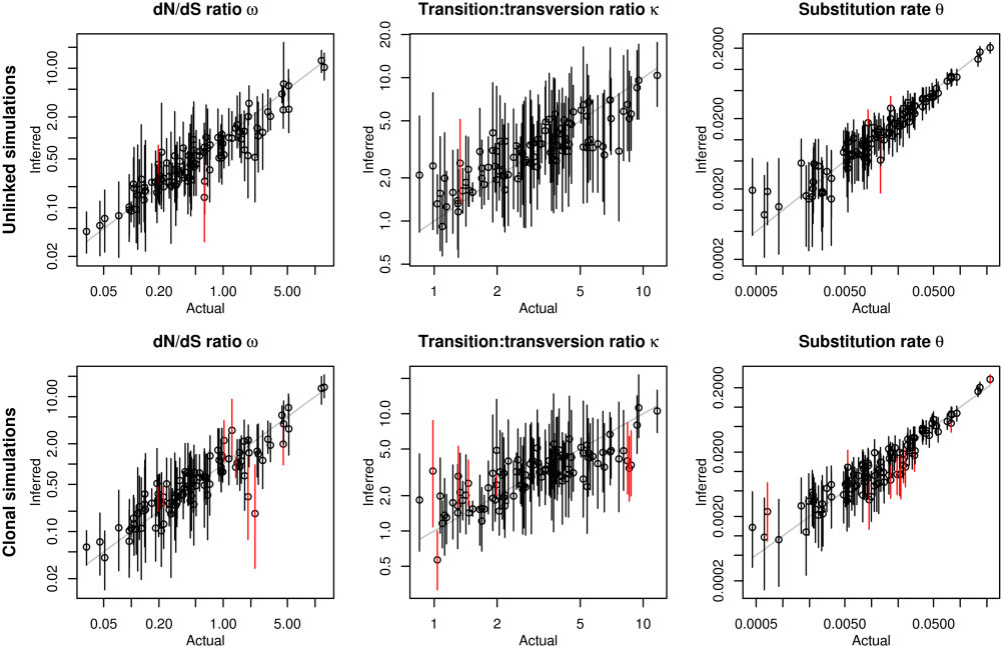
Performance of *genomegaMap* inference of *ω*, *κ*, and *θ* in simulations. In the Unlinked simulations (top row), every codon was simulated independently, favoring the *genomegaMap* assumption. In the Clonal simulations (bottom row), all codons were completely linked, disfavoring the *genomegaMap* assumption. Point estimates (posterior medians) and 95% credibility intervals are indicated by the circles and solid vertical lines, respectively, the latter colored red when they exclude the actual parameter. The number of simulations (out of 100) in which the 95% credibility intervals included the actual values of *ω*, *κ*, and *θ* were 98, 98, and 97 in the Unlinked simulations and 92, 92, and 88 in the Clonal simulations. The correlation between the point estimates and actual values of log ω,  log κ, and log θ were 0.86, 0.69, and 0.92 in the Unlinked simulations and 0.82, 0.61, and 0.88 in the Clonal simulations.

To test this claim more thoroughly, I evaluated the relative performance of *genomegaMap* in two scenarios. In the Unlinked simulations, 334 codons were simulated independently across 10,000 individuals, favoring the *genomegaMap* assumption. In the Clonal simulations, all codons were completely linked, strongly violating the *genomegaMap* assumption of unlinked sites. As expected, *genomegaMap* performed well in the Unlinked simulations, producing point estimates strongly correlated with the true values of the $d_{N}/d_{S}$ ratio *ω*, the transition:transversion ratio *κ* and the mutation rate *θ*, and 95% CIs that included the truth in 98%, 98% and 97%, respectively, of the 100 simulations (fig. 2).

In the Clonal simulations, codons were completely linked, maximally violating the independence assumption of *genomegaMap*. Despite this, the correlation between point estimates and true parameters remained strong, whereas the 95% CIs still included the truth in 92% of the 100 simulations for *ω* and *κ* and 88% of simulations for *θ* (fig. 2). These results suggest that *genomegaMap* produces only small loss in the accuracy of its point estimates and 95% CIs even when its independence assumption is completely wrong.

The major advantage of *genomegaMap* over *omegaMap* is its robustness to sample size. The computational run time of *omegaMap* increases with the square of the sample size. The run time of a comparable phylogenetic method would increase linearly with the sample size if the phylogeny were known; in practice co-estimating the phylogeny makes the computation much more intensive. In contrast, the run time of *genomegaMap* is constant with respect to sample size. This means it is uniquely suitable for the analysis of extremely large within-species data. To demonstrate its capabilities, I applied *genomegaMap* to 3,979 genes across 10,209 *M. tuberculosis* genomes.

### Characterizing Selection in 10,209 *M. tuberculosis* Genomes

*Mycobacterium tuberculosis* is a bacterial pathogen responsible for tuberculosis, one of the world’s leading causes of death. Twenty three percent of the global population is thought to carry latent infection, of whom 9.0–11.1 million people are estimated to have developed tuberculosis in 2017, with 1.5–1.7 million resulting deaths. Drug resistance is a major problem for tuberculosis treatment; an estimated 483,000–639,000 new cases were resistant to first-line drugs in 2017 (World Health Organization 2018).

The aim of the CRyPTIC Consortium is to help improve control of tuberculosis and facilitate better, faster and more targeted treatment of drug-resistant tuberculosis via genetic resistance prediction, paving the way toward universal drug susceptibility testing. CRyPTIC Consortium and 100,000 Genomes Project (2018) collected and whole-genome sequenced 10,209 *M. tuberculosis* genomes to quantify the performance of genomic prediction of drug resistance. The predictions were correct in 91.3–97.5% of resistant isolates and 93.6–99.0% of susceptible isolates for the four first-line drugs.

These predictions rely on existing knowledge of the genetic mechanisms of drug resistance. Vast data sets have the potential to reveal novel mechanisms of drug resistance through genome-wide association studies (GWAS). Such studies can benefit from an understanding of the selection pressures shaping genetic diversity and the identification of sites under positive selection because often that selection is driven by drug therapy (e.g., Farhat et al. 2013; Osório et al. 2013; Pepperell et al. 2013; Zhang et al. 2013; Lee et al. 2015; Koch et al. 2017; Mortimer et al. 2018).

**Fig. 3.**
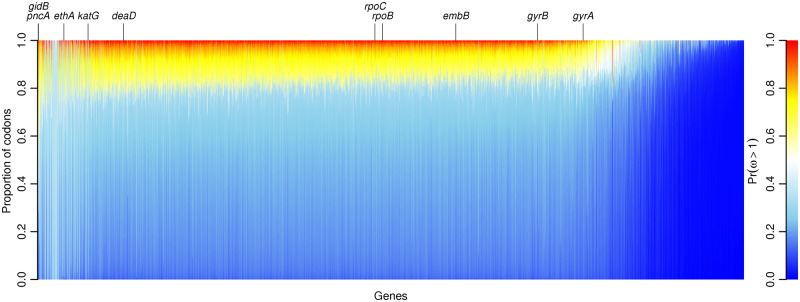
The evidence for positive selection across 3,979 genes in 10,209 *Mycobacterium tuberculosis* genomes. Each column is a stacked bar chart showing the proportion of codons in one gene with a given strength of evidence for positive selection, indicated by color. Blue indicates weakest evidence, Pr(ω>1)≈0, whereas red indicates strongest evidence, Pr(ω>1)≈1. Genes are ordered left-to-right by the mean Pr(ω>1) across codons, from highest to lowest. Notable genes containing codons with strong evidence of positive selection are labeled; these occur across the spectrum. The genes with predominantly sky blue color, scattered between *pncA* and *katG*, contained little information because they mapped poorly to the reference genome.

*Mycobacterium tuberculosis* is known for its complete lack, or near-complete lack, of homologous recombination (Godfroid et al. 2018), but as simulations showed, *genomegaMap* inference is robust to both recombination and the lack of recombination. I analyzed the 3,979 genes sequenced across the 10,209 genomes with *genomegaMap*. In 3,138 genes (79%), the model with independent *ω* for every codon fit better than the Bayesian sliding window model (supplementary table S1, Supplementary Material online). Figure 3 summarizes the evidence for positive selection across the genome by quantifying the posterior probability of ω>1. Most codons in most genes showed evidence against positive selection, that is, Pr(ω>1)<0.5, indicating functional constraint. In very few genes, such as *pncA* encoding pyrazinamidase, did positive selection appear to be more common. More often, the strongest evidence for positive selection was found in a small number of codons within genes dominated by negative selection, such as *gyrA*, encoding DNA gyrase subunit A. This shows how positive selection occurs against backdrops of both rapid amino acid change and functional constraint, so the mean Pr(ω>1) per gene provides limited insight.

Instead, I identified every gene with one or more codons exhibiting a posterior probability of positive selection of at least 90% (i.e., Pr(ω>1)≥0.9) (supplementary table S1, Supplementary Material online). The genes are annotated by their descriptions in GenBank and MycoBrowser (Kapopoulou et al. 2011). In total, 15,931/1,330,612 codons (1.2%) spanning 2,729/3,979 genes (69%) showed strong evidence of positive selection, a mean of 4.0 per gene. Among the most enriched for positively selected sites were genes encoding membrane proteins, toxin–antitoxin proteins (Sala et al. 2014), PE/PPE family proteins (Fishbein et al. 2015), ESX family proteins (Gröschel et al. 2016), and antimicrobial resistance (Farhat et al. 2013; Osório et al. 2013; Pepperell et al. 2013; Zhang et al. 2013; Lee et al. 2015; Koch et al. 2017; Mortimer et al. 2018).

### Positive Selection in Known Resistance-Determining Genes

**Fig. 4.**
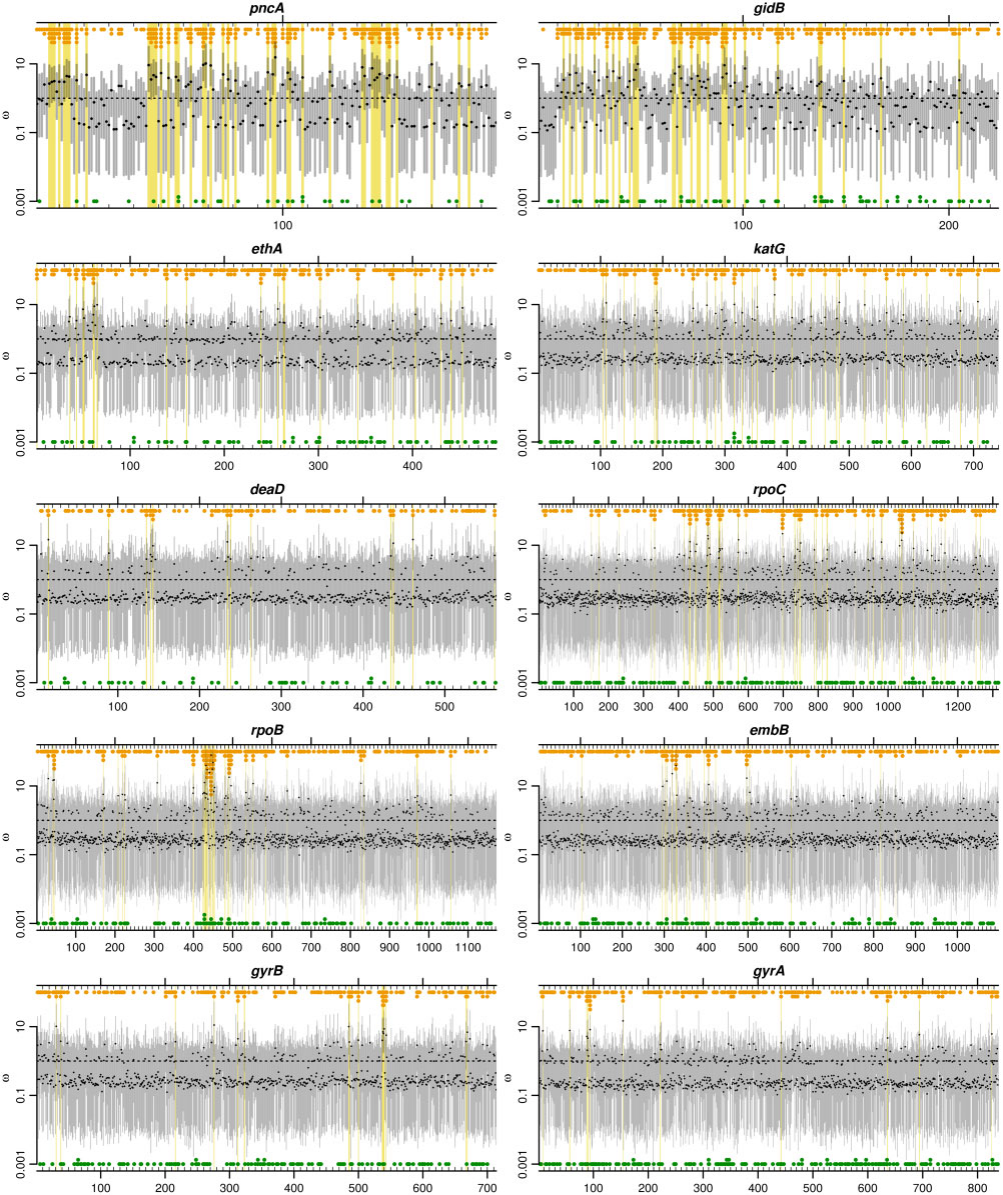
Evidence of positive selection in ten *Mycobacterium tuberculosis* genes across 10,209 genomes. Genes are ordered by the mean Pr(ω>1) across codons, from highest (*gidB*) to lowest (*gyrA*). Point estimates (black points) and 95% credibility intervals (gray bars) for *ω* are shown across codons. Codons for which Pr(ω>1)≥0.9 are highlighted with yellow boxes. Stacked points indicate the number of alleles that are nonsynonymous (orange) or synonymous (green) relative to the commonest allele.

Figure 4 shows in detail the variation in *ω* along ten genes, ordered by the mean Pr(ω>1) and cross-referenced above figure 3. In all ten genes, the model of independent *ω* for every codon fitted so much better than the Bayesian sliding window model that it dominated the results (100% posterior model probability, supplementary table S1, Supplementary Material online).

The signature of selection in *rpoB*, which encodes RNA polymerase subunit *β*, exemplifies the evolutionary response to antibiotic usage. Subunit *β* is targeted by the first-line drug rifampicin, which binds the RNA polymerase, interfering with transcription of DNA to mRNA (see e.g., Palomino and Martin 2014). Strong evidence of positive selection was found at 41 codons in *rpoB*, with a concentration of 15 in a 28-codon hotspot covering codons 427–454 coinciding with the *rifampicin resistance determining region* and including the common serine-to-leucine substitution at position 450 (S450L; positions relative to NC_000962.2). The population harbors a large number of alternative amino acid alleles in this region, represented by an accumulation of orange points in figure 4; this provides the signature of elevated $d_{N}/d_{S}$. The extremely large sample size greatly enhances the ability to discover these alternative alleles, many of which are rare. For example, codon 445, which showed the highest point estimate of ω=79.8, harbors 14 alleles encoding 12 different amino acids, with H445Y the most abundant amino acid substitution at only 1.5% frequency. Additional signals were observed including codons 45, 399–400 and 491. None of these sites is included in the WHO-endorsed GeneXpert MTB/RIF assay despite evidence of involvement in MDR-TB outbreaks (e.g., Makhado et al. 2018). For exhaustive results at the codon level, see https://doi.org/10.6084/m9.figshare.10329311.

The adjacent *rpoC* gene, encoding RNA polymerase subunit β′, showed similar peaks of positive selection against a backdrop of constraint. 51 codons showed strong evidence of positive selection, including codons 434, 483–485, 491, 515–519, 698, and 1039–1040. Several of these regions coincide with high-probability compensatory mutations identified by Comas et al. (2012): P434R, V483A/G, D485H/N, I491T/V, and N698H/K/S. The compensatory mutations mitigate the fitness deficit imposed on rifampicin-resistant *M. tuberculosis* by mutations in the rifampicin resistance determining region of *rpoB*. These positions localize to the interface between RNA polymerase subunits *α* and β′, suggesting they play a role in the interaction between subunits (Comas et al. 2012). The extremely large sample size revealed other rare amino acid alleles at these positions that could also be compensatory: D485Y and N698D/L.

The World Health Organization (2018) report that 82% of rifampicin-resistant tuberculosis cases are also resistant to the first-line drug isoniazid, making them multidrug resistant tuberculosis (MDR-TB), which requires longer treatment with more toxic drugs. Isoniazid is a prodrug requiring activation by catalase-peroxidase, encoded by *katG*. In an earlier draft of the article, where the results were based solely on a Bayesian sliding window analysis, *genomegaMap* did not detect evidence of positive selection surpassing the posterior probability threshold of 90% in *katG*. This was puzzling because *katG* displayed the highest level of homoplasy (an indicator of positive selection) among 23 resistance-associated genes in an earlier study of 2,099 genomes (Walker et al. 2015). Upon reanalysis, the independent *ω* per codon model fitted much better than the Bayesian sliding window model (100% posterior probability) and picked out strong evidence of positive selection at 28 codons in *katG*. They included the resistance-conferring S315T substitution, which (Walker et al. 2015, supplementary fig. S17, Supplementary Material online) found emerged 180 times. Intense selection for an individual mutation has been characterized as a “tight target” by Mortimer et al. (2018). *GenomegaMap* does not exploit the signal of homoplasy to infer positive selection because it does not use a phylogenetic tree, relying instead on the relative number of nonsynonymous alleles. Nevertheless, the posterior probability of positive selection at codon 315 was 100% in the new analysis.

Resistance to the first-line drug ethambutol is conferred by mutations in *embB*, which encodes an essential part of the cell wall biosynthetic pathway (Palomino and Martin 2014). Selection is predominantly conservative in *embB*. Against this background, 16 codons were found to exhibit strong evidence of position selection, including D328F/G/H/I/F and M306I/L/V, which has been implicated in ethambutol resistance, Q497H/K/P/R and Y319C/D/S.

The DNA gyrase-encoding genes *gyrA* and *gyrB* displayed strong signatures of positive selection localized to the quinolone resistance determining regions, surrounded by constraint characteristic of essential proteins. Eleven and sixteen codons, respectively, reached the 90% probability threshold, including codons 88, 90, and 94 in *gyrA* and 537–540 in *gyrB*. Several of these positions are known to confer resistance to second-line quinolone drugs, including *gyrA* A90E/G/V and D94A/G/H/N/Y (Palomino and Martin 2014).

Selection at *ethA*, which encodes a nonessential monooxygenase, bore a similar profile of selection to *katG* (fig. 3), whose product is also nonessential. Loss-of-function mutations in *ethA* prevent activation by monooxygenase of the second-line ethionamide from a prodrug to its active form (Palomino and Martin 2014). Strong evidence for positive selection was apparent at 21 codons in *ethA*, including pairs of codons at positions 50–51, 61–62, and 262–264. Like *katG*, this suggests that although resistance-conferring loss-of-function mutations could occur throughout the gene, they tend not to. The selection regimes of *ethA* and *katG* presumably reflect a balance between antimicrobial-imposed positive selection for loss-of-function mutations conflicting with functional constraint favoring conservation of the gene products.

Rapidly evolving genes dominated by positive selection are rare in *M. tuberculosis*, and when they do occur they are perhaps exemplified by *pncA*. Whereas 44/186 codons (24%) showed strong evidence of positive selection, this signal is driven by probable loss-of-function mutants, making it a particular form of positive selection that adapts the organism by disrupting protein function. The *pncA* gene encodes the nonessential enzyme pyrazinamidase, which converts the first-line prodrug pyrazinamide to its active form. Resistance to pyrazinamide is achieved by loss-of-function mutations in *pncA* (Palomino and Martin 2014). Function-ablating missense and nonsense mutations have spread rapidly in response to the widespread use of pyrazinamide. The regions where evidence for positive selection is weaker may be under stronger functional constraint in environments where expression of the gene is favored.

The *gidB* gene shows strong evidence of positive selection at 31/224 codons (14%) scattered throughout most of its length. This gene encodes a methyltransferase that increases resistance to the second-line drug streptomycin. Streptomycin inhibits protein synthesis by binding to the 16S rRNA component of the 30S ribosomal subunit, increasing mistranslation. Loss-of-function of the *gidB* methyltransferase is thought to alter methylation of a highly conserved 16S rRNA residue, preventing binding by streptomycin (Okamoto et al. 2007; Wong et al. 2011). Like in *pncA*, this mechanism creates a selection pressure favoring missense and nonsense mutations throughout the gene, a phenomenon characterized as a “sloppy target” by Mortimer et al. (2018). However, the modest increase in resistance conferred by this mechanism and the current status of streptomycin as a relatively less-frequently used, second-line drug with strong side effects suggests there may be other selection pressures driving *gidB* loss-of-function.

### Positive Selection in a Cold-Shock Protein

I scanned the *genomegaMap* results for evidence of positive selection at genes in which the selective forces driving adaptation are unknown or incompletely understood. In particular, I looked for genes with the characteristic signature of positive selection against a backdrop of functional constraint. The *deaD* gene, encoding cold-shock DEAD-box protein A and also known as *csdA*, is one such example (fig. 4), with strong evidence of positive selection at 13/563 codons (2.3%).

DEAD-box proteins are a large family of ATP-dependent RNA helicase proteins found in prokaryotes and eukaryotes that separate double-stranded RNA molecules in an energy-dependent manner. They are named after their highly conserved Asp-Glu-Ala-Asp (D-E-A-D) motif. DEAD-box proteins are involved in ribosome biogenesis, translation initiation and RNA decay, fundamental processes that must dynamically respond to changes in environment and stress (Linder and Fuller-Pace 2013).

In *Escherichia coli*, the DeaD/CsdA protein has been characterized as essential for ribosome formation during cold shock because it separates stable secondary RNA structures which form at low temperature (Jones et al. 1996). DeaD/CsdA is important for biogenesis of both the 30S and 50S ribosome subunits, conferring tolerance toward mutants of other regulators and ribosomal proteins (Moll et al. 2002; Charollais et al. 2004). DeaD/CsdA has also been found to control gene expression at temperatures relevant to the mammalian host, and for modulating the carbon storage regulatory (Csr) system, which globally regulates mRNA translation and turnover (Vakulskas et al. 2014).

Strong evidence of positive selection in *M. tuberculosis deaD* was evident at codons 140 and 143, with weaker evidence of positive selection at four of the five other codons in the region 139–145 (Pr(ω>1)≥0.65). This region, which encodes TPGRMID, corresponds to motif Ib, consensus sequence TPGRXXD, one of a series of highly conserved motifs that characterize DEAD-box proteins. Motif Ib overlaps a nine-residue alpha helix (α7) beginning at codon 140 in *M. tuberculosis*. Sengoku et al. (2006) characterized the structure of the *Drosophila melanogaster* DEAD-box protein Vasa in detail. They found that two RecA-like domains in the DEAD-box protein core bind a single RNA strand and sharply bend it. The bend avoids a clash between the RNA and a “wedge” formed by α7 when the RNA is single stranded, whereas the unbound strand of an RNA duplex would be predicted to clash with the α7 wedge, resulting in disrupted base-pairing.

The residues homologous to four codons in motif Ib directly interact with the bound RNA (Sengoku et al. 2006). These positions exhibited a single alternative amino acid allele each across the 10,209 genomes: T139P, G141D, R142P, and D145H. The two positions with strong evidence of positive selection—P140L/S and M143I/R/V—exhibited multiple alternative amino acid alleles, whereas I144 was invariant. No synonymous variation was seen across the motif. Despite the relatively abundant amino acid variation in the motif in terms of allele numbers, the frequency of all substitutions except M143I/R/V was extremely low, <0.5%. The sensitivity of the $d_{N}/d_{S}$ ratio to allele numbers, irrespective of allele frequencies, was observed earlier in *rpoB*. The diversity of rare alleles could mirror the mode of selection in the *rpoB* rifampicin resistance determining region, in which any of a large collection of amino acid substitutions improve fitness in the presence of the drug.

The DEAD-box motif itself, covering codons 163–166 and responsible for RNA binding, ATP binding and interdomain interactions, was situated in a region of conservation, with a mean probability of positive selection of 22%. This, together with the general conservation throughout the gene, suggests that the effect of substitutions in motif Ib might not be to knock out the function of DeaD, but to modify it in some way; for instance, by altering conformation in such a way as to change interactions with other molecules.

Given the functional characterization of DeaD, candidate drivers of adaptation in motif Ib may in some way inhibit ribosome biogenesis or translation by interfering with ribosomal proteins, rRNAs or amino acids through mutation, for example with reactive oxygen radicals produced by the immune response, conformational change, for example binding by an antibiotic, or changes in molecular availability, for example caused by nutrient deprivation, cold shock or other stress. In the case of drug resistance, the detection of localized positive selection against a backdrop of constraint in *deaD* provides valuable context for future GWAS searching for genetic variants responsible for the growing problem of drug resistant infections.

## Discussion

The main advantages of *genomegaMap* for estimating $d_{N}/d_{S}$ ratios within species are 1) it is fast no matter how large the sample size and 2) it accounts for recombination. These advantages were achieved by extending the Wilson et al. (2011) approximation to the distribution of allele frequencies under parent-dependent mutation models, and assuming independence between codons. Simulations showed good performance despite these approximations.

Among the benefits of the approach, haplotype information is not required and missing data are easily handled, making *genomegaMap* suitable for short-read exome and genome sequencing data. The *genomegaMap* approach is to treat $d_{N}/d_{S}$ as a substitution parameter. In this light, it can be seen as a general, likelihood-based method for estimating substitution parameters within species under parent-dependent mutation models.

The approach has several limitations. Sites are assumed independent between codons but linked within codons. Despite this, simulations showed good performance when recombination was high and low. Thus, it was possible to analyze 10,209 genomes from *M. tuberculosis*, an almost perfectly clonal organism. The effects of violating other assumptions including constant population size, no population structure and random sampling were not investigated. The importance of sampling cannot be overstated, with signatures of selection entirely dependent on the selection pressures experienced by the populations analyzed.

Perhaps the greatest limitation of *genomegaMap* is its use of the $d_{N}/d_{S}$ ratio to characterize natural selection. Within species, $d_{N}/d_{S}$ is expected to vary even in a constant environment, with ratios closer to one expected for younger variants not yet exposed to selection for so long (McDonald and Kreitman 1991). Further, the form of positive selection that best predicts a high $d_{N}/d_{S}$ ratio is diversifying selection, in which any amino acid is favored over the incumbent. Diversifying selection may be relatively limited, to arms races, for example, between host and pathogen, or to heterogeneous environments, for example, immunologically diverse hosts. The evolution of resistance to antibiotics since their introduction in the 1940s may resemble such a Red Queen scenario assuming fitness trade-offs, because exposure varies from host-to-host.

Examples from *rpoB* and *deaD* showed that the signal of elevated $d_{N}/d_{S}$ stems mainly from the abundance of alternative amino acid alleles, relative to the number expected under neutrality, and not from allele frequencies. Some of these alternative alleles were detected at frequencies <0.5%, demonstrating the value of extremely large sample sizes. The sliding window model employed by *genomegaMap* fit the data worse than an independent *ω* model for most coding sequences (CDSs), but smaller samples may benefit from its smoothing effect.

Interpreting $d_{N}/d_{S}$ within species has been criticized by proponents of process-driven models. Models of $d_{N}/d_{S}$ within species are essentially descriptive. They formally treat selection like mutational bias, where differences in nonsynonymous and synonymous diversity arise purely from different mutation rates. In contrast, process-driven models explicitly parameterize selection coefficients separately from mutation rates. When the model assumptions are valid, the inferred selection coefficients are more interpretable than $d_{N}/d_{S}$. But a major assumption is that selection at multiple sites interacts simply, for example, by reducing effective population size. When linkage disequilibrium is low, simulations may support this (e.g., Wilson et al. 2011). When it is high, as in many pathogen genomes, forces like clonal interference challenge the validity of single-site selection models. In which case it may be preferable to embrace the descriptive $d_{N}/d_{S}$ approach rather than interpret the parameters of a misspecified process-driven model. Power to detect forms of adaptation that do not markedly elevate $d_{N}/d_{S}$ is inevitably limited (Kryazhimskiy and Plotkin 2008), but genuine signals of $d_{N}/d_{S}\gg 1$ are usually of biological interest.

Despite its limitations, the relatively simple interpretation of $d_{N}/d_{S}$ ratios means the approach continues to hold a strong appeal. For such applications, *genomegaMap* helps accelerate the exploitation of big data for gaining new insights into evolution within species.

## Supplementary Material

msaa069_Supplementary_Data

## Appendix A: Members of the CRyPTIC Consortium

Derrick W. Crook, Timothy E.A. Peto, A. Sarah Walker, Sarah J. Hoosdally, Ana L. Gibertoni Cruz, Joshua Carter, Clara Grazian, Sarah G. Earle, Samaneh Kouchaki, Alexander Lachapelle, Yang Yang, David A. Clifton, and Philip W. Fowler, University of Oxford; Zamin Iqbal, Martin Hunt, and Jeffrey Knaggs, European Bioinformatics Institute; E. Grace Smith, Priti Rathod, Lisa Jarrett, and Daniela Matias, Public Health England, Birmingham; Daniela M. Cirillo, Emanuele Borroni, Simone Battaglia, Arash Ghodousi, Andrea Spitaleri, and Andrea Cabibbe, Emerging Bacterial Pathogens Unit, IRCCS San Raffaele Scientific Institute, Milan; Sabira Tahseen, National Tuberculosis Control Program Pakistan, Islamabad; Kayzad Nilgiriwala and Sanchi Shah, The Foundation for Medical Research, Mumbai; Camilla Rodrigues, Priti Kambli, Utkarsha Surve, and Rukhsar Khot, P.D. Hinduja National Hospital and Medical Research Centre, Mumbai; Stefan Niemann, Thomas A. Kohl, and Matthias Merker, Research Center Borstel; Harald Hoffmann, Katharina Todt, and Sara Plesnik, Institute of Microbiology & Laboratory Medicine, IML Red, Gauting; Nazir Ismail, Shaheed Vally Omar, and Lavania Joseph, National Institute for Communicable Diseases, Johannesburg; Guy Thwaites, Thuong Nguyen Thuy Thuong, Nhung Hoang Ngoc, Vijay Srinivasan, and Timothy M. Walker, Oxford University Clinical Research Unit, Ho Chi Minh City; David Moore, Jorge Coronel and Walter Solano, London School of Hygiene and Tropical Medicine and Universidad Peruana Cayetano Heredá, Lima; George F. Gao, Guangxue He, Yanlin Zhao, and Chunfa Liu, China CDC, Beijing; Aijing Ma, Shenzhen Third People’s Hospital, Shenzhen; Baoli Zhu, Institute of Microbiology, CAS, Beijing; Ian Laurenson and Pauline Claxton, Scottish Mycobacteria Reference Laboratory, Edinburgh; Anastasia Koch, Robert Wilkinson, University of Cape Town; Ajit Lalvani, Imperial College London; James Posey, CDC Atlanta; Jennifer Gardy, University of British Columbia; Jim Werngren, Public Health Agency of Sweden; Nicholas Paton, National University of Singapore; Ruwen Jou, Mei-Hua Wu, Wan-Hsuan Lin, CDC Taiwan; Lucilaine Ferrazoli, Rosangela Siqueira de Oliveira, Institute Adolfo Lutz, São Paulo. Authors contributing to the CRyPTIC Consortium are (in alphabetical order): Irena Arandjelovic (Institute of Microbiology and Immunology, Faculty of Medicine, University of Belgrade, Belgrade, Serbia), Angkana Chaiprasert (Faculty of Medicine Siriraj Hospital, Mahidol University, Thailand), Iñaki Comas (Instituto de Biomedicina de Valencia [IBV-CSIC], Calle Jaime Roig, Valencia, Spain; FISABIO Public Health, Valencia, Spain; CIBER in Epidemiology and Public Health, Madrid, Spain), Francis A. Drobniewski (Imperial College, London, UK), Maha R. Farhat (Harvard Medical School, Boston, USA), Qian Gao (Shanghai Medical College, Fudan University, Shanghai, China), Rick Ong Twee Hee (Saw Swee Hock School of Public Health, National University of Singapore, Singapore), Vitali Sintchenko (Centre for Infectious Diseases and Microbiology—Public Health, University of Sydney, Sydney, Australia), Philip Supply (Univ. Lille, CNRS, Inserm, CHU Lille, Institut Pasteur de Lille, U1019—UMR 8204—CIIL—Centre d’Infection et d’Immunité de Lille, F-59000 Lille, France), and Dick van Soolingen (National Institute for Public Health and the Environment [RIVM], Bilthoven, The Netherlands).
